# Marchiafava-Bignami Disease in a Nonalcoholic Diabetic Patient

**DOI:** 10.1155/2013/979383

**Published:** 2013-04-24

**Authors:** Sisira Yadala, Jin Jun Luo

**Affiliations:** ^1^Department of Neurology, Temple University School of Medicine, 3401 North Broad Street, Suite C525, Philadelphia, PA 19140, USA; ^2^Department of Pharmacology, Temple University School of Medicine, Philadelphia, PA 19140, USA

## Abstract

Marchiafava-Bignami disease (MBD) is a rare neurological disorder mostly seen in alcoholic and malnourished patients with a pathognomonic hallmark of corpus callosum demyelination. MBD in nonalcoholics without malnutrition has rarely been reported. We report a case of MBD in a diabetic patient, without alcoholism or malnutrition, caused by a wide range of glycemic level fluctuations. A 38-year-old man presented with sudden onset of alteration in speech and multiple falls in three days. Neurologic examination showed dysarthria, dysmetria, and ataxia but, otherwise, normal cranial nerves, motor and sensory functions, and tendon reflexes. Brain MRI showed symmetric abnormalities in the splenium of the corpus callosum. In addition, demyelination was also observed in bilateral posterior limbs of the internal capsule and brachium ponti. His symptoms significantly improved after stabilization and normalization of his plasma glucose level and administration of multivitamins and corticosteroids. The underlying pathophysiology of the development of MBD in our case is likely to be osmotic stress from a wide range of glycemic fluctuations causing structural and functional disturbance of oligodendrocytes, which may be reversible in its early stage.

## 1. Introduction

Marchiafava-Bignami disease (MBD) is a rare neurodegenerative disorder with a pathognomonic hallmark of corpus callosum demyelination [1]. It was first reported in Italian wine drinkers about 110 years ago [2]. Clinical presentation may vary from mild impairment in consciousness, gait abnormalities, dysarthria, pyramidal signs, seizures, neuropsychiatric, and behavioral alterations to stupor and coma [1]. MBD has mostly been reported in the subjects with chronic heavy alcohol consumption and malnourishment. However, there is paucity of MBD in nonalcoholics in the literature.

## 2. Case Report

A 38-year-old man presented with acute onset of dysarthria and gait disequilibrium resulting in multiple falls in three days. He had a past medical history of diabetes, bipolar disorder, and depression. The records of his home blood-glucose measurements disclosed his plasma glucose levels fluctuated frequently from 30 to 450 mg/dL in the prior three months. He was well nourished without a history of alcoholism. Neurological examination showed normal language but significant dysarthria with bizarre phonation, loss of prosody, slow articulation, and imprecise consonant utterance. Finger-to-nose and heel-knee-shin testing were dysmetric bilaterally. His gait was strikingly ataxic and wide based. The remaining neurologic examination of cranial nerves, motor and sensory functions, and reflexes was normal.

**Figure 1:**
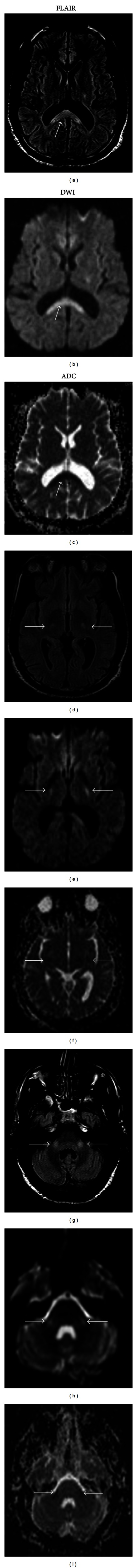
Brain MRI of fluid attenuated inversion recovery (FLAIR), diffusion-weighted imaging (DWI), and apparent diffusion coefficient(ADC) images showing altered attenuation (arrow) in splenium of corpus callosum (a–c), bilateral posterior limbs of internal capsule (d–f), and bilateral brachium ponti (g–i).

Comprehensive laboratory studies at admission showed an increased plasma glucose (214 mg/dL, normal: 65–110), glycosylated hemoglobin (8.4%, normal: 4.7–5.7), erythrocyte sedimentation rate (34 mm/h, normal: 0–10), and C-reactive protein (25.1 mg/L, normal: <0.8) but normal hemogram, electrolytes, lipids, thyroid, renal, and liver functions. Tests for HIV, *syphilis,* hepatitis, sarcoidosis, and Lyme disease were all negative. Cerebrospinal fluid study showed increased glucose (164 mg/dL, normal: 50–80), protein (64 mg/dL, normal: 15–40), and IgG (7.1 mg/dL, normal, 0.5–6.1): but normal cell counts, myelin basic protein, smears, cultures, and viral tests. Oligoclonal bands were absent. Toxicology screen was unrevealing. Conventional somatosensory, visual, and brainstem evoked potentials were normal. Brain MRI showed altered strong signals within the splenium of corpus callosum and symmetrically mild intensities in bilateral posterior limbs of the internal capsules and brachium ponti (Figure 1). The findings suggested a central nervous system demyelinating disorder, consistent with MBD [1].

The patient was treated with hydration, blood glucose stabilization, intravenous methylprednisolone (1000 mg daily for 3 days), and multivitamins. His speech, dexterity, and gait were subsequently improved in 5 days. His diabetic regimen was adjusted, and he was discharged to rehabilitation. One month later, in a follow-up visit, only a mildly ataxic gait was noted. Complete recovery was reported by telephone after nine months.

## 3. Discussion

MBD is a rare neurologic entity typically seen in alcoholics and malnourished patients. MBD in a nonalcoholic is very rare while in nonalcoholic diabetics is even rarer [3–5]. To our knowledge, our case is the second MBD in a nonalcoholic diabetic patient. 

Recently, Suzuki and colleagues reported the first nonalcoholic diabetic MBD case of a 37-year-old woman [6]. In contrast to Suzuki's patient who had untreated and persistently elevated serum glucose and isolated lesion in the corpus callosum [6], our patient had a wide range of unstable and fluctuations of glycemic levels and abnormal neuroimaging findings showing altered signals involving the splenium of the corpus callosum and extracallosal white matter pathways (Figure 1). The pathophysiology of corpus callosum demyelination in MBD is not completely understood. Wide ranges of glycemic fluctuation with frequent hyperglycemia and hypoglycemia in alternation may cause osmotic stress on oligodendroglial cells, provoking structural and functional aberrance, similarly as seen in central pontine myelinolysis [7]. The appearance of abnormal signals on diffuse-weighted images corresponding to those on the apparent diffusion coefficient images suggested the possibility that energy production disarrangement occurred in those neural structures. The pattern of symmetric distribution of the lesions on brain MRI (Figure 1) strongly suggested an etiology of metabolic origin, likely due to the wide ranges of glycemic fluctuation with frequent hyper- and hypoglycemic alternations. 

Advanced technology such as MRI has facilitated evaluation of the corpus callosum which makes premortem diagnosis of MBD feasible [8, 9]. However, the usefulness of MRI in predicting the prognosis of MBD is limited. Early studies suggested that factors such as rate and severity of clinical progression and lesions in callosal versus extracallosal structures may have prognostic values [1, 8, 9]. Subsequently, those notions have been challenged because acute and subacute MBD cases do not necessarily have a fatal outcome [10] but have survived and even made a good recovery with only mild sequelae within a period of few months [11]. Additionally, improvement after symptomatic treatment was observed in our patient. Thus, it seems incorrect to assign it a prognostic value on the basis of extracallosal involvement on MRI findings [12]. 

Treatment of MBD was usually empirically initiated with multivitamins [1, 6, 13], corticosteroids [6, 14, 15], stabilization of plasma glucose [6], and supportive care. Early correct diagnosis and prompt appropriate management may be critical in reversing the underlying pathophysiology in the early stage.
